# Comparative evaluation of interdental papilla fills around bone level and tissue level implants: A randomised controlled trial

**DOI:** 10.6026/973206300222332

**Published:** 2026-04-30

**Authors:** Aishwarya Rajendra Mulay, Ashok Kumar Bhansali, Maya S Indurkar, Dhalkari C.D, Pawan Kumar, Madhu Varma

**Affiliations:** 1 Department of Periodontology, Government Dental College and Hospital, Chhatrapati Sambhajinagar, Maharashtra, India

**Keywords:** Dental implants, interdental papilla, tissue-level implants, bone-level implants, soft tissue aesthetics, single-tooth replacement

## Abstract

Early achievement of optimal interdental papilla fill around dental implants remains a clinical challenge affecting aesthetic outcomes in single-tooth replacement. Therefore, it is of interest to compare interdental papilla fill around bone-level and tissue-level implants in patients undergoing single-tooth replacement. Twenty systemically healthy patients were randomly allocated into two groups: bone-level implants (n=10) and tissue-level implants (n=10). Tissue-level implants demonstrated significantly greater mesial papilla fill at 3 months compared to bone-level implants (p=0.022), while no significant difference was observed in distal papilla fill (p=0.19). Thus, we show that tissue-level implants may provide improved early papilla formation, although long-term clinical relevance requires further evaluation.

## Background:

The ideology of success in dental implant treatment has been changing significantly in the last 20 years. Although the basic biological condition of implant survival is the presence of ossuaroik impedance, the modern standards of the treatment are now focusing on the result of aesthetics as an essential determinant of the success of a treatment [1]. The placement of implant-supported restorations in continuity with the adjacent natural dentition is something that must be done with great caution to the soft tissue architecture especially interdental papilla that is central in creating a pleasing gingivism [2]. Interdental papilla is an intricate anatomical formation that is either present or absent and hence plays a major role in the aesthetic appearance of the implant therapy. The loss or decrease of papillary tissue leads to the development of so called black triangle which is invariably ranked among the most unappealing aesthetically consequences of implant dentistry [3]. Studies have revealed that patients will notice even small changes in papilla morphology and even shortcomings in the form of 2-3 millimeters are perceptible to both professionals and amateurs [4]. Numerous factors determine the development and sustenance of interdental papillae of dental implants. They are the horizontal and vertical distance between the adjacent teeth and implants, the status of bone crest of the interproximal bone, the soft tissue biotype, emergence profile of the prosthesis and the design features of the implant system used [5]. The connection between levels of crestal bone and overlaying soft tissue is amongst these variables, which have been widely investigated [6]. Both bone-level and tissue-level dental implant systems are widely classified into either systems and each has different design features which can affect the peri-implant soft tissue behaviour. The bone-level implants are placed to have the platform at the alveolar crest or slightly lower, which means that they need an abutment connection after implantation and may also have to be done in two-stage surgery [7]. Conversely, tissue-based implants include a machined transmucosal collar which places the restorative platform about 1.5-3.0 mm above the crestal bone which in many cases leads to single-step surgical procedures [8]. The bone crest-implant-abutment interface vertical placement has been cited as the origin of marginal bone remodelling effects. It has been indicated that when this interface is placed in supracrestal position, which is naturally attained with tissue level designs, crestal bone loss may be reduced relative to that experienced when it is either subcrestal or crestal as is common with bone level systems [9]. This is a pattern of differential bone remodelling, which, in theory, may have an effect on the dimensions of overlying soft tissue, such as papilla height [10]. Although there is a lot of literature studying the marginal bone variation of various types of implants, there are relatively fewer studies that have been conducted in interdental papilla formation as a primary outcome measure. The current literature shows contradictory results on the impact of implant design on the papillary outcomes and methodological heterogeneity of the literature restricts comparison of results across studies. Moreover, the majority of evidence found relates to research in anterior maxillary areas and there is scarcity of evidence in posterior areas [11]. The correlation between bone level alterations and soft tissue dimensions indicate that the pattern of designing implants that affect bone retention can also increase the formation of papilla. Theoretical benefits of placing the microgap outside of crestal bone could be achieved by tissue-level implants [12]. Therefore, it is of interest to report the interdental papilla fill around-bone and tissue-level single-tooth implants, which were placed in the posterior areas of the jaw, with baseline and three months postoperative outcome measures.

## Materials and Methods:

## Design and ethical concerns of the study:

The department of periodontology in the Government dental college and hospital was the place where this prospective randomized controlled trial was done in the year 2025. The approval of the study protocol was obtained at the Institutional Ethics Committee before starting the recruitment of the participants. The trial was reported and planned in line with the Consolidated Standards of Reporting Trials (CONSORT) of randomized controlled trial. All participants signed the informed consent written after the study procedures, possible risks and benefits were explained to them in detail.

## Sample size determination:

The calculation of sample size was done using the previous literature that had investigated outcomes of interdental papilla around dental implantation. Based on the assumption of a desired effect size of 0.8, alpha error of 0.05 and statistical power of 80, the required number of participants per group was calculated as 10.

## Participant selection:

Single tooth edentulous spaces in the posterior maxilla or mandible were recruited by taking 20 patients who presented to the outpatient department. Eligibility criteria used to enroll the participants were as follows:

## Inclusion criteria:

[1] Age between 18 and 65 years

[2] Systemically healthy individuals

[3] Presence of a single posterior tooth edentulous space

[4] Adequate bone volume for implant placement without augmentation procedures

[5] Sufficient soft tissue quality and quantity

[6] Healthy periodontium with probing depths ≤3 mm on adjacent teeth

[7] Willingness to comply with follow-up protocols

[8] Provision of written informed consent

## Exclusion criteria:

[1] Uncontrolled systemic diseases including diabetes mellitus

[2] Requirement for guided bone regeneration procedures

[3] Current tobacco use

[4] Pregnancy or lactation

[5] History of radiotherapy or chemotherapy to the head and neck region

[6] Long-term corticosteroid therapy

[7] Active periodontal disease

[8] Inadequate oral hygiene compliance

## Randomization and allocation concealment:

Following baseline assessment and confirmation of eligibility, participants were randomly allocated to one of two treatment groups using a coin toss method. Allocation concealment was maintained through sequentially numbered, opaque, sealed envelopes prepared by an investigator not involved in participant enrollment or treatment delivery.

[1] Group A (n=10): Bone-level implant placement

[2] Group B (n=10): Tissue-level implant placement

## Baseline assessment:

All participants were taken through comprehensive clinical and radiographic assessments before the intervention. The demographic information such as age, sex and medical history was observed. The clinical evaluation involved the evaluation of the interdental papilla sizes with a calibration periodontal probe (Hu-Friedy Colorvue Probe, Chicago, USA). The measurements were taken at the tip of the interdental papilla to the cementoenamel junction (CEJ) of the adjacent teeth at the mesial and the distal sides. The computed tomography cone-beam (CBCT) was conducted to assess the bone height, width and quality that were available at the proposed location of the implant.

## Surgical protocol:

A single experienced periodontist carried out all the surgical operations to reduce inter-operator error and facilitate consistency in treatment delivery.

[1] Group A (Bone-Level Implant): 2% lidocaine with 1:100000 epinephrine was used as a local anesthetic. An incision was made in the middle of the crestals with a number 15 surgical blade. The flaps of the mucoperiosteal were raised to reveal the alveolar ridge. Sequential drilling was done in line with the procedure of the manufacturer to prepare the site of osteotomy. An implant was inserted at crestal level with a bone-level implant (Osstem TS III, Osstem Implant Co., South Korea). An immediate healing abutment was immediately attached and primary wound closure was done with 4 0 silk sutures.

[2] Group B (Tissue-Level Implant): The operation was similar to that of Group A. After flap deepening and sequential osteotomy preparation, a tissue-level implant (Osstem SS III, Osstem Implant Co., South Korea) was inserted with the smooth collar being placed transmucosally. A cover was applied and primary closure was done using 4-0 silk sutures. Every contender was given standardized postoperative protocols and antibiotic prophylaxis (amoxicillin 500mg thrice daily during 5 days) and pain killers (ibuprofen 400 mg on demand). Chlorhexidine 0.12 per cent mouthwash was given on two-week prescription. All the implants were not loaded with prosthetics throughout the study.

## Outcome measurements:

Interdental papilla fill was the primary measure of outcome that was evaluated on the mesial and distal sides relative to the place where the implant was. These measurements were taken at two points: base line (just before the surgery) and three months after the surgery. A periodontal probe was calibrated and the vertical distance between the tip of the interdental papilla and the CEJ of the adjacent natural tooth measured. Measurements were done by one calibrated examiner who was not aware of group assignment.

## Follow-up protocol:

The patients would be followed up in one week and three months after the implant placement. Removal of sutures was done at the one week visit. There was clinical examination during every visit which was in terms of healing, assessment of complications and reinforcement of oral hygiene guidance.

## Statistical analysis:

Data examination was done in the Statistical Package of Social Sciences (SPSS) version 26.0. Descriptive statistics were in the form of mean SD of the continuous variables and frequencies in percentages of the categorical variables. The test of normality of data distribution was conducted using the Shapiro Wilk test. The Mann-Whitney U test was used to compare groups on the basis of non-normal variables because of non-normality. The Wilcoxon signed-rank test was used to do within-group comparisons across time points. In an appropriate way, the Chi-square test or the Fisher exact test was used to compare categorical variables. A p value of p<0.05 was set as statistically significant.

## Results:

The demographic characteristics of study participants are presented in (Table 1 - see PDF). The mean age of participants in Group A (bone-level implants) was 43.10 ± 8.90 years (range: 30-60 years), while participants in Group B (tissue-level implants) had a mean age of 41.30 ± 4.20 years (range: 35-48 years). Statistical analysis revealed no significant difference in age distribution between the two groups (t=0.58, p=0.57). The gender distribution across study groups is summarized in (Table 2 - see PDF). In Group A, 60% (n=6) of participants were female and 40% (n=4) were male. Group B demonstrated an equal gender distribution with 50% (n=5) female and 50% (n=5) male participants. Overall, the study population comprised 55% females and 45% males. Chi-square analysis indicated no statistically significant difference in gender distribution between groups (χ^2^=0.20, p=0.65). Comparative analysis of interdental papilla dimensions between study groups is presented in (Table 3 - see PDF). No statistically significant differences were observed between groups for either mesial or distal papilla measurements at baseline. The mean mesial papilla height was 2.58 ± 0.77 mm in Group A and 2.60 ± 0.43 mm in Group B (Z=0.34, p=0.73). Similarly, baseline distal measurements were comparable between groups (Group A: 2.40 ± 0.54 mm; Group B: 2.48 ± 0.62 mm; Z=0.52, p=0.60). At the three-month follow-up, the tissue-level implant group demonstrated significantly greater mesial papilla height compared to the bone-level group (3.64 ± 0.55 mm versus 3.22 ± 0.69 mm; Z=2.29, p=0.022). However, distal papilla measurements did not differ significantly between groups at this time point (Group A: 3.02 ± 0.71 mm; Group B: 3.32 ± 0.64 mm; Z=1.32, p=0.19). Analysis of the change from baseline to three months revealed significantly greater mesial papilla fill in the tissue-level implant group compared to the bone-level group (1.04 ± 0.52 mm versus 0.64 ± 0.49 mm; Z=2.24, p=0.025). The difference in distal papilla change between groups did not reach statistical significance (Group A: 0.62 ± 0.56 mm; Group B: 0.84 ± 0.53 mm; Z=1.41, p=0.16). No major complications were observed in either group during the study period. All implants achieved primary stability at placement and no implant failures occurred. Minor postoperative swelling and discomfort were reported by participants in both groups, which resolved within the expected timeframe following standard postoperative care.

## Discussion:

The current randomized controlled trial examined the interdental papilla formation surrounding single-tooth implants of either type, at the bone level and tissue level, at the posterior part of the jaw. The results have shown that at three months postoperative, there was a significant difference in mesial papilla fill on tissue-level implants than the bone-level implants, but not on the distal aspect. The enhanced mesial papilla complex using tissue-level implants accords with the theoretical benefits of supracrestal placement of implant- abutment interface. The studies have confirmed that placement of microgap between implant and abutment affects the crestal bone remodelling and a more coronal positioning could probably diminish inflammatory stimuli on the bone level [13]. The transmucosal collar structure of tissue-level implants places this critical junction out of the crestal bone that could be more spared in the initial healing period [14]. The importance of the vertical distance between the bone crest and the contact point in determining papilla fill has been repeatedly determined in investigations examining the dimensions of soft tissues around a single-tooth implant. It has been shown that with this distance more than 5 millimetres, the entire regeneration of papilla is more erratic [15, 16]. The current results indicate that implant design could be another adjustable variable affecting such a relationship, possibly, via its impact on marginal bone stability. The observation of the substantial variation in the mesial but not the distal aspect is worth attending. Past studies have indicated asymmetrical papilla formation around dental implants with a number of studies indicating less papilla dimensions on the distal surface than on the mesial [17]. This can be explained by anatomical reasons that involve the position of the adjacent tooth, such as being either root-diameter different or proximal contour of the cementoenamel junction and connective tissue attachment features [18]. Also, this asymmetry could be caused by differences in the accessibility of oral hygiene to mesial and distal surfaces. Comparative studies that have studied the marginal bone loss in bone-level versus tissue- level implants have had uneven results. Other studies have documented reduced early bone loss at the bone-level implants and others have been able to show benefits over time with tissue-level designs [19]. Marginal bone changes and soft tissue outcomes have a complicated relationship and they might not be proportional. The three-month follow-up of the present study embodies initial healing dynamics, which could not be similar to the dynamics in the long-term. Direct linear assessment of the papilla formation in this study was conducted instead of categorical index like the Jemt papilla fill index. Although the categorical system of scoring provides simplicity and reliability when using inter-examiner, the method might not be sensitive enough to detect subtle variation in the papilla dimensions. Direct millimetric measurements are continuous data that can be more suitable in the detection of treatment effects in controlled clinical trials [20]. The clinical value of statistically significant differences in papilla height should, however, be interpreted with caution because the small differences might not be noticeable by patients or clinicians. The studies of the factors affecting interdental papilla surrounding implants have shown that there are various determinants of this factor besides the design of the implants. The distance between the implant and the adjacent tooth in the horizontal direction is normally advised to be kept at 1.5-2.0 millimeters; this influences the possibility of the formation of papillas [21]. In the same way, the periodontal attachment conditions of neighboring natural teeth have a great impact on interproximal soft tissues. Research has proved that it is more predictable when neighboring teeth have a healthy periodontal support [22]. The sequence of placement of the implants in comparison to the extraction of the teeth has also been researched on as a possible predictor of soft tissue results. The systematic reviews investigating this association have discovered that although instant and deferral location procedures can present comparable results as far as papilla preservation is concerned, individual specific elements and surgical approach still hold significance as predictors [23]. The current experiment has incorporated only healed extraction sites, therefore, holding this variable constant and permitting the separation of effects of implant designs. Another important consideration that affects the outcome of the peri-implant tissue is the soft tissue biotype. It has been concluded that thick gingivial biotypes are linked to more positive soft tissue responses to dental implants such as increased papilla formation and decreased risk of recession [24]. Although in the current study only patients with sufficient quality of soft tissues have been taken into consideration, no particular biotype categorization has been carried out, which is also one of the possible confounding factors. The surgical procedure that has been adopted in implant uncovering has been proven to affect the results (the papilla) in two stage procedures. Different countermeasures to traditional crestal incisions have been suggested to increase papilla formation such as methods that save or add interproximal soft tissues [25]. A one stage surgical procedure with immediate abutment placement was applied in the current research that can be seen as having merits on preserving soft tissue structure by preventing a second surgical intervention [26]. The current research has a number of limitations that need to be mentioned. The sample size is relatively small, which may not be able to statistically detect fine differences, especially on the distal aspect. The 3-month follow-up period though sufficient in measuring early healing patterns does not allow measuring the long-term papilla stability. Moreover, functional restoration effects on the size of papillas were not evaluated since the study period was not accompanied by loading the prosthetic. The limitation to posterior location, although providing uniformity, will restrict generalizability to anterior aesthetic areas where papilla results can be of more clinical relevance. These findings must be interpreted with caution with regard to their clinical implications. Tissue-level implants showed statistically significant better mesial papilla formation, but the difference (about 0.4 mm) might be not reflected into any clinical or aesthetic benefits. However, the type of an implant design can be one adjustable variable that should be considered in a case when the maximum preservation of papilla is expected. Investigations in the future must also include higher sample sizes, longer follow-up phases and replace the unused implant designs with prosthetically restored ones to obtain more detailed information on how the implant design influences the outcome of interdental papilla. Moreover, the study of anterior locations where aesthetics are of utmost importance would make the findings even more clinical.

## Conclusion:

Tissue-level implants demonstrated improved early mesial interdental papilla fill compared to bone-level implants, suggesting a potential advantage in achieving favorable aesthetic outcomes. No significant differences were observed in distal papilla formation between the two designs. Implant selection, along with appropriate preoperative assessment, plays an important role in optimizing peri-implant soft tissue aesthetics.
